# Assessing the impact of global versus local ancestry in association studies

**DOI:** 10.1186/1753-6561-3-s7-s107

**Published:** 2009-12-15

**Authors:** Sun Jung Kang, Emma K Larkin, Yeunjoo Song, Jill Barnholtz-Sloan, Dan Baechle, Tao Feng, Xiaofeng Zhu

**Affiliations:** 1 Department of Epidemiology and Biostatistics, Division of Genetic and Molecular Epidemiology, Case Western Reserve University School of Medicine, Wolstein Research Building, 2103 Cornell Road, Cleveland, Ohio 44106 USA; 2 Center for Clinical Investigation, Case Western Reserve University School of Medicine, Wolstein Research Building, 2103 Cornell Road, Cleveland, Ohio 44106 USA; 3 Case Comprehensive Cancer Center, Case Western Reserve University School of Medicine, 11100 Euclid Avenue, Cleveland, Ohio 44106 USA

## Abstract

**Background:**

To account for population stratification in association studies, principal-components analysis is often performed on single-nucleotide polymorphisms (SNPs) across the genome. Here, we use Framingham Heart Study (FHS) Genetic Analysis Workshop 16 data to compare the performance of local ancestry adjustment for population stratification based on principal components (PCs) estimated from SNPs in a local chromosomal region with global ancestry adjustment based on PCs estimated from genome-wide SNPs.

**Methods:**

Standardized height residuals from unrelated adults from the FHS Offspring Cohort were averaged from longitudinal data. PCs of SNP genotype data were calculated to represent individual's ancestry either 1) globally using all SNPs across the genome or 2) locally using SNPs in adjacent 20-Mbp regions within each chromosome. We assessed the extent to which there were differences in association studies of height depending on whether PCs for global, local, or both global and local ancestry were included as covariates.

**Results:**

The correlations between local and global PCs were low (*r *< 0.12), suggesting variability between local and global ancestry estimates. Genome-wide association tests without any ancestry adjustment demonstrated an inflated type I error rate that decreased with adjustment for local ancestry, global ancestry, or both. A known spurious association was replicated for SNPs within the lactase gene, and this false-positive association was abolished by adjustment with local or global ancestry PCs.

**Conclusion:**

Population stratification is a potential source of bias in this seemingly homogenous FHS population. However, local and global PCs derived from SNPs appear to provide adequate information about ancestry.

## Background

Association studies, whether genome-wide or candidate-gene-based, have emerged as a popular tool to identify underlying genetic variants with small disease-related effects. One commonly cited limitation of these studies is that undetected population stratification (PS) can cause false-positive and/or false-negative findings, and therefore proper analysis should be applied in these studies [1,2].

Genome-wide association studies that adjust for PS using information from markers selected across the entire genome address the effect of global PS, which is mainly driven by the demographic history of a population. In contrast, local genome regions harboring functional variants may be subject to subtle forms of PS not only because of demographic history but also due to natural selection and random fluctuations of admixture. In addition to admixture from different continents, PS can also present in populations from the same continent [3], as demonstrated by the association of the lactase gene (*LCT*) and adult height in a European-American population [4], which is driven by the North-South admixture in European populations. Here we use the Framingham Heart Study (FHS) data from the Genetic Analysis Workshop 16 (GAW16) to assess the differences in genome-wide association studies of height using adjustment for global versus local ancestry to account for PS.

## Methods

The 500 k single-nucleotide polymorphism (SNP) data from the Framingham Offspring Cohort of GAW16 Problem 2 was used for analysis. We selected unrelated adults (ages 21-60) from each family (i.e., spouses) based on an algorithm that prioritized individuals with higher genotyping rates, selecting individuals at random when needed. Height was regressed on age and age squared across all visits separately for each sex, with the resultant average standardized residuals used as the outcome of interest. SNPs with greater than 10% of calls missing and with minor allele frequencies less than 1% were excluded from analysis.

Principal components (PCs) of marker genotype data were calculated to represent an individual's ancestry using the program FamCC [5]. Global ancestry is characterized by 10 PCs derived from all SNPs across the genome regardless of linkage disequilibrium (LD) between the markers. Similarly, local ancestry is based on 10 PCs derived from SNPs in non-overlapping 20-Mbp regions in the genome, yielding 141 distinct regions. To determine how much of each local PC could be explained by the set of global PCs, we regressed all 10 global PCs against each of the 10 local PCs and computed the percent of variation in the local PC that could be explained by the full set of global PCs. Furthermore, we calculated the mean correlation statistics between global PCs (1 through 10) and corresponding local PCs (1 through 10). To compare ancestry adjustment methods, we tested three groups of covariates in regression models: 1) ten global PCs, 2) ten local PCs in a local region where a testing SNP is located, or 3) both global and local PCs. Each test SNP was coded additively. To examine the effect of using correlated SNPs, we compared the local PCs obtained from region containing the *LCT *gene using: all SNPs (ignoring LD structure), every other SNP in a 20-cM region and SNPs with *r*^2 ^< 0.5. We also compared our results with SNPs in common with three research articles that conducted genome-wide association studies with height in a variety of European-American populations [6-8].

## Results

### Global and local principal components

The number of SNPs in each local 20-Mbp region ranged from a minimum of 898 to a maximum 5,016, with the majority of regions falling between 2,000 and 4,000 SNPs. The average adjusted *r*^2 ^value estimated by regressing the first local PC on all global PCs is 0.05 ± 0.12. The global PCs explain more than 15% of the variability of the first local PC for only 5 of the 141 first local PCs. The proportion of variation explained by global PCs decreases for the next two to ten local PCs to less than 0.03. The mean correlation between the global first PC and local first PC is 0.11 ± 0.10. The mean correlations between the other global PCs and the local PCs are lower and range from 0.025 to 0.050, suggesting that the first local and global PCs share the most ancestral information or North-South European ancestry.

### Distribution of p-values for association study of height allowing for ancestry

**Figure 1:**
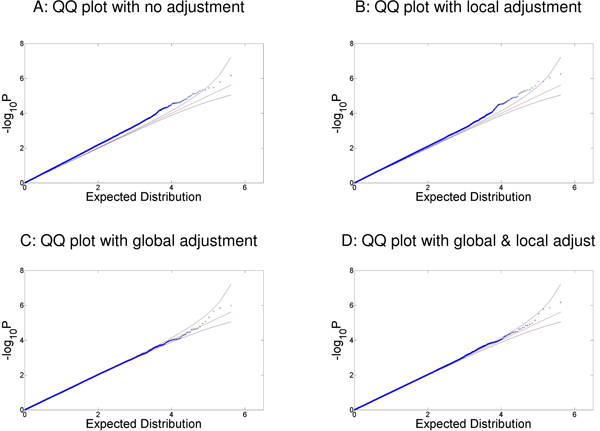
**Q-Q Plots comparing *p*-values for the association between SNPs and height to the uniform distribution for different ancestry estimates**. Figures depict the Q-Q plots comparing (A) the -log(unadjusted *p*-values); (B)-log(local adjusted /*p*/-values) (C) -log(global adjusted /*p*/-values) and (D) -log(both local and global adjusted *p*-values) to the theoretical uniform distribution.

Figure 1 presents the quartile-quartile (Q-Q) plots which compare the distribution of the *p*-values in association tests between height and all SNPs against the expected uniform distribution of *p*-values under the null. Figure 1A shows that the *p*-values unadjusted for ancestry have an inflated type 1 error rate (inflation factor = 1.11). Figure 1B suggests that adjusting for local ancestry alone with 10 PCs can reasonably control the effect of population stratification (inflation factor = 1.07). Adjusting for global ancestry by 10 PCs (inflation factor = 1.01) and both global and local ancestry with all 20 PCs (inflation factor = 1.02), as shown in Figure 1C and 1D, suggests results most consistent with the uniform distribution under the null.

Table 1 presents the summary statistics for the rank correlations between the Wald test statistic for the association between the test SNP and height after including 10 global PCs and the Wald test statistic after including the 10 local PCs. The rank correlations are averaged across all regions and presented as mean, standard deviation, and minimum and maximum rank correlation observed. Results are also presented with Wald test statistics that consider both global and local ancestry PCs as covariates. The rank correlations are less than one and therefore suggest that global and local ancestry estimates contain different information. The correlations become even smaller when restricting to SNPs with *p *< 0.05 (for either Wald test statistic), suggesting that the test statistic in the tail of the distribution may be affected more severely by whether global or local ancestry adjustment is used.

**Table 1:** Mean rank correlations between Wald test statistics after adjusting for 10 global PCs (global), 10 local PCs (local), or both global and local PCs (both)

Wald test Statistic^a^					
				
1	2	SNPs	Mean rank correlation (SD)^b^	Minimum	Maximum
Global	Local	all SNPs	0.72 (0.04)	0.50	0.81
Global	Local	*p *< 0.05	0.60 (0.07)	0.35	0.72
Global	Both	all SNPs	0.82 (0.05)	0.58	0.92
Global	Both	*p *< 0.05	0.71 (0.09)	0.35	0.87
Local	Both	all SNPs	0.81(0.02)	0.75	0.88
Local	Both	*p *< 0.05	0.68 (0.05)	0.53	0.80

^a^The Wald test statistic is calculated as the β coefficient/standard error for the relationship between the test SNP and height, after adjusting for the PCs as indicated in the column.

^b^The rank correlation is calculated between the two Wald test statistics in each local region and then averaged using all SNPs in the dataset or only the SNPs with *p *< 0.05 for either the first or second Wald test statistics.

### SNPs within the lactase gene

In Table 2, we present the results of the three methods of ancestry adjustment for the top three significant SNPs in the *LCT *gene. Unadjusted analyses show significant associations for many SNPs in the *LCT *gene; while adjusting for local, global, or both results in the disappearance of the association evidence. Local PCs based on all SNPs or a subset of SNPs suggest that using all SNPs for PC estimation provides excellent adjustment for ancestry by removing all significant associations for these top SNPs. The differences in adjusted *p*-values between the various local SNP selections are less dramatic for SNPs not presented. The correlations between the local PC1 values derived from these methods are >0.92, suggesting little impact on the choice of SNPs for local PC estimation.

**Table 2:** *p*-Values for the three most significant SNPs located within the lactase gene, by varying ancestry adjustment methods

			*p*-Value local adjusted^b^				
					
Chr	SNP^a^	*p*-Value unadjusted	All SNPs	Every other SNP	SNPs *r*^2 ^< 0.5	*p*-Value global adjusted	*p*-Value local^c ^and global adjusted
2	RS2322660	0.00008	0.99	0.80	0.31	0.25	0.81
2	RS2322659	0.00043	0.80	0.70	0.30	0.21	0.56
2	RS2015532	0.00411	0.81	0.91	0.47	0.35	0.83

^a^SNPs: RS1042712, RS3769013, RS3769012, RS872151, RS2304371, RS4954445 were all significant at *p *< 0.05 in unadjusted models and adjustment for ancestry by any method resulted in all *p *> 0.35. SNP RS11884924 within the lactase gene was not significant *p *> 0.45 for all association tests.

^b^Local PCs are estimated for different combinations of SNPs: 1) all local SNPs (*n *= 2773); 2) every other SNP (*n *= 1386) or 3) SNPs with *r*^2 ^< 0.5 (*n *= 969).

^c^Local PC is estimated from all SNPs in a region.

### SNPs associated with height in published genome-wide association studies

Of the 112 SNPs presented in the main tables from each of three research articles that had previously conducted a genome-wide association study of height [6-8], only 40 SNPs were found in the FHS 500 k SNP panel. The *p*-values observed for these SNPs are presented in Table 3 for associations with height for any *p*-value < 0.10 using the different ancestry adjustment methods. In general, the results for adjusting for global and local ancestries are similar, except for a handful of SNPs for which local ancestry adjustment is less significant than unadjusted models (RS2562784, RS8041863, and RS7846385) or SNPs for which global ancestry adjustment is less significant than unadjusted models (RS7153027, RS1390401, and RS6686842). Adjusting for both local and global ancestries provides comparable results to local and global ancestry adjustment conducted individually.

**Table 3:** Significant SNPs (*p *< 0.10) for height observed in three published papers available in the Framingham 500 k panel (sorted by unadjusted *p*-values)

			*p*-Value			
			
Chr	Reference	SNP^a^	unadjusted	Local adjusted	Global adjusted	Local & global adjusted
4	[6]	RS1812175	0.0001	0.0011	0.0003	0.0012
12	[8]	RS11107116	0.0058	0.0187	0.0046	0.0198
15	[6]	RS4533267	0.0306	0.0064	0.0413	0.0123
15	[7]	RS2562784	0.0359	0.1486	0.0497	0.0942
20	[6]	RS967417	0.0374	0.0270	0.0226	0.0156
3	[8]	RS6440003	0.0475	0.0632	0.0658	0.0661
13	[8]	RS3116602	0.0487	0.0515	0.0351	0.0878
14	[6]	RS7153027	0.0575	0.0537	0.7278	0.9571
15	[8]	RS8041863	0.0584	0.1346	0.0721	0.1374
1	[8]	RS1390401	0.0898	0.0333	0.8022	0.8150
8	[6]	RS7846385	0.1417	0.3020	0.0459	0.0874
1	[8]	RS6686842	0.1715	0.0552	0.3438	0.1440
6	[8]	RS4549631	0.2195	0.0738	0.2099	0.1399
15	[8]	RS10906982	0.2231	0.1135	0.0115	0.0179

^a^SNPs rs6060369, rs6060373, rs11858942, rs6854783, rs2055059, rs2282978, rs31198, rs6724465, rs12735613, rs10958476, rs4794665, rs3791675, rs10935120, rs1042725, rs6088792, rs2916448, rs749052, rs4743034, rs8099594, rs2814993, rs16896068, rs2814828, rs798544, rs12199222, rs6830062, and rs10512248 have *p *> 0.10 for all unadjusted and adjusted results and are not reported here.

## Discussion

One way to control population stratification is to use markers distributed across the genome, which reflects demographic history of a population. Nevertheless, smaller regions that actually harbor disease-causing variants may be subject to demographic history, natural selection pressure, or random fluctuations in admixture. Thus, it becomes important to look at local ancestry in local regions of the chromosomes, where causal variants may exist to better control for population stratification. In the FHS sample, estimates of global ancestry do not explain local ancestry PCs, as illustrated by low adjusted *r*^2 ^values from the regression models. The low correlations observed between the global and local PCs further suggest that estimates of global and local ancestry each provide different information about ancestry.

We observed that the distribution of *p*-values adjusting for either global, or both local and global ancestries can best fit the expected distribution under the null, while adjusting for local ancestry slightly departs from the null distribution. It is known that there are many variants underlying height, which has an estimated heritability of 0.8 in world populations. Three recent genome-wide association studies uncovered over 50 independent SNPs for height cumulatively accounting for approximately 2-4% of the variation per study [6-8], suggesting many more remaining variants. We would expect some degree of departure of the observed *p*-value distribution from the null, although further investigation is required to assess the degree of this departure. Many of the SNPs either are causal or in LD with causal variants, and can contribute the departure from the null distribution of *p*-values adjusting for local ancestry, although detection requires much larger sample sizes.

The effectiveness of adjusting for local ancestry can be observed from the results of the association analysis between height and SNPs in *LCT *gene, known to be a false-positive finding. All three ancestry adjustment methods can remove the spurious association observed in unadjusted analyses. The decrease in significance for the top SNPs is much more substantial when using local ancestry or both global and local ancestry compared with global ancestry alone, indicating that incorporating local ancestry estimates can adequately control the effect of population stratification.

In theory, the best way to adjust for population stratification is to use the true ancestry at the testing locus. However, this true ancestry at the locus is difficult to estimate, especially for a population formed from several ancestral populations. The local PCs estimated the SNPs around the locus may better approximate the ancestry at the locus than the global PCs estimated using the SNPs across the genome. However, the number of SNPs that are required to adequately estimate the ancestry at a locus needs further investigation. In the *LCT *gene we demonstrate that selecting less correlated SNPs does not affect estimation of local PCs, and for the most significant *LCT *SNPs, using the maximal number of SNPs in a local region may provide the best reduction in spurious association.

This analysis does not explore whether varying the size of the local PC region would alter the ancestry estimates or change the observed associations between significant SNPs and height. We also do not explore the optimal number of PCs to account for ancestry, whether local or global, and perhaps there is additional loss of power with extraneous PCs in the model. Certainly, the first global and local PCs are the most correlated and capture most of the ancestral information; however, adjusting for both local and global ancestry may result in a statistical over adjustment and potential loss of statistical power. Previous studies suggest that the largest 10 PCs are able to capture the ancestry in most of the current world populations [9]. We also note that studying a population with more stratification (i.e., African-Americans) may offer more insight into the comparison of global versus local ancestry adjustment.

## Conclusion

These findings suggest that adjusting for local ancestry may control the false-positive rate due to the effect of population stratification in the FHS sample. Further analysis with known causal variants or on simulated data will be necessary to determine which method of PC adjustment for ancestry is the most powerful, while maintaining proper type 1 error.

## List of abbreviations used

FHS: Framingham Heart Study; GAW16: Genetic Analysis Workshop 16; LD: Linkage disequilibrium; PC: Principal components; PS: Population stratification; Q-Q: Quartile-quartile; SNP: Single-nucleotide polymorphism.

## Competing interests

The authors declare that they have no competing interests.

## Authors' contributions

EKL, SJK, and XZ drafted the manuscript. EKL and SJK analyzed the data. YS, DB, and TF provided essential computer programming for data analysis. XZ conceived of the idea and provided critical oversight of the analyses. JB-S helped interpret the data and edited the manuscript. All authors read and approved the manuscript.
